# Atypical Fibroxanthoma-Like Amelanotic Melanoma: A Diagnostic Challenge

**DOI:** 10.3390/dermatopathology8010004

**Published:** 2021-01-12

**Authors:** Gerardo Cazzato, Anna Colagrande, Antonella Cimmino, Giovanni Liguori, Teresa Lettini, Gabriella Serio, Giuseppe Ingravallo, Andrea Marzullo

**Affiliations:** 1 Section of Pathology, Department of Emergency and Organ Transplantation, University of Bari, 70121 Bari, Italy; gerycazzato@hotmail.it (G.C.); anna.colagrande@gmail.com (A.C.); micasucci@inwind.it (A.C.); gabriella.serio1@uniba.it (G.S.); giuseppe.ingravallo@uniba.it (G.I.); andrea.marzullo@uniba.it (A.M.); 2 Section of Dermatology, Department of Biomedical Science and Human Oncology, University of Bari, 70121 Bari, Italy; giovanni.liguori@policlinico.ba.it

**Keywords:** melanoma, skin, neoplasm, fibroxanthoma

## Abstract

Atypical fibroxanthoma-like amelanotic melanoma is a very rare variant of melanoma that can, if not correctly recognized and framed, lead to diagnostic errors that can potentially cause problems of extreme relevance to patients. Correct knowledge of this entity and the execution of adequate immunohistochemical investigations are the basic conditions for the correct management of this lesion. We report on a case of atypical fibroxanthoma-like amelanotic melanoma, which clinically simulated a fibrohistiocytic lesion, and which created differential diagnostic problems, and finally, we conduct a short review of the literature.

## 1. Introduction

Atypical fibroxanthoma-like amelanotic melanoma is a very rare form of melanoma described in the literature [1,2,3] that creates many problems in terms of differential diagnosis. In particular, this melanoma can mimic many skin cancers and benign skin lesions [3], and its correct diagnosis is of fundamental importance for the nosographic classification of the lesion, its correct oncological management, and therefore, the best possible outcome for the patient. We report on a rare case of melanoma with the morphological characteristics of atypical fibroxanthoma (AFX) that clinically resembled a fibrohistiocytic lesion and that caused diagnostic problems in our practice. We also review the current literature with particular emphasis on the clues that can reduce the possibility of, if not avoid, diagnostic error.

## 2. Case Report

**Figure 1:**
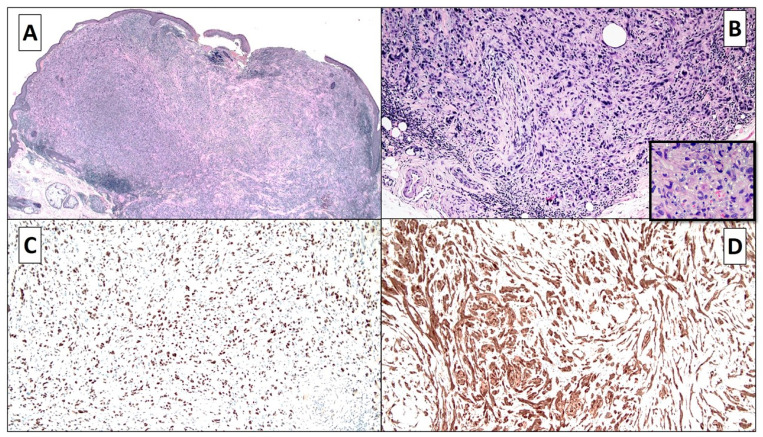
(**A**,**B**) The lesion was centered in the dermis, ulcerated, and composed of epithelioid and some spindle cells with pleomorphic, hyperchromatic, and sometimes nucleolated nuclei, with moderate to abundant eosinophilic cytoplasm and arranged in a fascicular and sometimes random fashion. In (**B)**, the presence of giant multinucleated cells and the involvement of a nerve are also appreciated (hematoxylin and eosin, original magnification, 40×–100×). (**C**,**D**) Immunostainings for SOX-10 and S-100 proteins were highly positive in cancerous components, including in the giant multinucleated cells (SOX-10 and S-100 proteins, original magnifications 100×).

A 93-year-old man came to the University Dermatology and Venereology Department following the identification of a lesion to the right auricle of approximately 1 cm in diameter that had been present for about 2 years. The lesion had been interpreted by the dermatologist as a benign fibrohistiocytic tumor without suspicious dermoscopical appearance. The lesion was excised, formalin-fixed, and paraffin-embedded. Histopathological examination of hematoxylin and eosin-stained sections revealed a lesion centered in the dermis, ulcerated, and composed of epithelioid and sometimes spindle cells with pleomorphic, hyperchromatic, and nucleolated nuclei, with moderate to abundant eosinophilic cytoplasm and arranged in a fascicular and occasionally random fashion (Figure 1A). There were also giant multinucleated cells, especially in the superficial part of the lesion. At a higher magnification, both typical and atypical mitotic figures were observed. There were clusters of peritumoral inflammatory lymphocytic infiltration (Figure 1A,B). Phenomena such as necrosis and hemorrhage were not evident. There was no melanin pigment present, and the dermoepidermal junction did not present any junctional melanocytic growth, without significant alterations. The lesion was noted to make contact with the nerves, surrounding them, without evidence of invasion (Figure 1B).

Based on these morphological features, the differential diagnosis included, initially, sarcomatoid squamous cell carcinoma, or atypical fibrohistiocytic lesion, or finally, a variant of melanoma. Therefore, some immunohistochemical tests were carried out in order to confirm this clinical and pathological diagnosis.

The immunohistochemical tests for p40, p63, vimentin, CD34, and CD31 were negative (not shown), and immunostaining for CD68 was focally positive in rare giant cells. Immunostainings for SOX-10 and S-100 proteins were strongly positive, including in some giant cells (Figure 1C,D), and S-100 protein also stained some nerves present at the base of the lesion.

Reactions for melan-A and HMB-45 were negative (not shown). Immunostaining for CD10 (positive in many AFXs) was strongly positive.

Based on the strong SOX-10 and S-100 expressions, the lesion was diagnosed as melanoma with morphological features of AFX, measuring to a Breslow depth of 3.1 mm and a Clark level of IV. The patient subsequently underwent PD-1 immunotherapy.

## 3. Discussion

Melanoma is one of the most aggressive malignancies [3,4]. Its correct identification and consequent diagnosis is of fundamental importance in order to correctly manage the patient. Unconventional melanomas can mimic many different tumors, such as dermatofibrosarcoma protuberans, malignant fibrous histiocytoma (MFH), myxofibrosarcoma (myxoid MFH), malignant hemangiopericytoma, and malignant schwannoma, which leads to many problems for the pathologist [3,4,5]. In particular, we describe a case of a very rare variant of melanoma with morphological characteristics similar to those of atypical fibroxanthoma. This variant has very rarely been described in the literature, with only five cases found in research published on PubMed and Medline [1,2,3,4,5,6]. More specifically, five cases reported by various authors have been described [1,2,3,4], and their findings are summarized in Table 1. All patients were old (>65 years), and AFX–melanoma developed in parts of the body exposed to sunlight (mainly head and neck). It should be emphasized that in two cases, the clinical suggestion did not take into account the possibility of melanoma. In two previously reported cases, melanocytic immunohistochemical markers (S-100, melan-A, HMB-45) were completely negative. In contrast, our case demonstrated strong reactivity to SOX-10 and S-100, leading us to consider a melanocytic lesion. Another very important aspect consists in the fact that the lesion in our case had large ulceration, making it impossible to identify an area of melanocytic junctional proliferation that would have helped the diagnosis of melanoma, also using hematoxylin and eosin. In addition to strong S-100 and SOX-10 positivity, there were small-caliber nerves with associated mast cells, features that have been described in neurotropic/desmoplastic-type melanomas [7]. While the combination of S-100/SOX-10 positivity and CD68 negativity could also suggest a malignant peripheral nerve sheath tumor, these lesions tend to occur deeper in the dermis or subcutis compared with melanoma. Although, morphologically, the lesion was consistent with a diagnosis of AFX, negativity for CD-10, CD68 (PGM-1), and vimentin was not in agreement with such a diagnosis. Recently, CD10 expression has been reported in sarcomatoid undifferentiated melanoma [8]. Because the lesion was ulcerated and a junctional component could not be evaluated, differentiating between a primary and a metastatic lesion was not possible. However, imaging studies (CT/MRI) did not reveal tumors in other areas of the body.

## 4. Conclusions

In conclusion, we have described the sixth rare case of AFX-like amelanotic malignant melanoma, which is a very rare entity that can be difficult to diagnose. Pathologists should be aware of this rare melanoma variant and utilize appropriate immunohistochemical testing to avoid a diagnostic pitfall.

## Figures and Tables

**Table 1:** Atypical fibroxanthoma-like melanoma: clinical–pathological findings of five cases reported and the present case.

No.	Age (y)/Gender	Clinical Manifestation	Immunohistochemistry Study	Thickness (Breslow)	Reference
1	70/M	Two exophytic lesions: left cheek and forehead	Both lesions: S-100 protein: positive, HMB-45: negative	Left cheek: 3.1 mm Forehead: 1.3 mm	Sangueza et al. [3]
2	88/F	Amelanotic lesion (1 cm): left forearm	S-100 protein, HMB-45, and melan-A/MART1: positive	Not available	Lee et Al. [2]
3	67/F	Ulcerated nodule in the center of a pigmented macule	S-100 protein, HMB-45, and melan-A/MART1: positive CD68: positive in dendritic cells	2.5 mm	Sangueza et al. [3]
4	80/M	Basal cell carcinoma?	S-100 protein, HMB-45, and melan-A/MART1: negative CD68: positive for xanthomatous macrophages and foreign body giant cells	Not available	Sangueza et al. [3]
5	72/F	Two separate erythematous nodules within a large hypopigmented patch: left cheek	S-100 protein, HMB-45, and melan-A/MART1: positive CD68: focally positive CD163: negative	3.4 mm	Chao-Kai Hsu et al. [1]
6	93/M	Nodule on the right auricle	S-100 protein, SOX-10: positive melan-A, HMB-45: negative CD68: focally positive	3.1 mm	Cazzato et al.
